# S1‐Leitlinie Tinea capitis

**DOI:** 10.1111/ddg.70395

**Published:** 2026-10-05

**Authors:** Peter Mayser, Dietrich Abeck, Philipp P. Bosshard, Jochen Brasch, Georg Daeschlein, Isaak Effendy, Yvonne Gräser, Peter Höger, Julia Huynh, Maria Kinberger, Annette Kolb‐Mäurer, Katharina Antonia Langen, Bartosz Malisiewicz, Alexander Nast, Hagen Ott, Christin Pelzer, Dieter Reinel, Martin Schaller, Silke Uhrlaß, Pietro Nenoff

**Affiliations:** ^1^ Biebertal Deutschland; ^2^ Hautarztpraxis am Tibarg Hamburg Deutschland; ^3^ Dermatologische Klinik Universitätsspital Zürich Universität Zürich Zürich Schweiz; ^4^ Klinik für Dermatologie, Venerologie und Allergologie, Mykologisches Labor Universitätsklinikum Schleswig‐Holstein, Campus Kiel Kiel Deutschland; ^5^ Medizinische Hochschule Brandenburg, Medizinische Fachhochschule Anhalt/ Köthen Köthen Deutschland; ^6^ MVZ – Dermatologie Franziskus Hospital Bielefeld Bielefeld Deutschland; ^7^ Charité – Universitätsmedizin Berlin, Korporatives Mitglied der Freien Universität Berlin und Humboldt‐Universität zu Berlin, Institut für Mikrobiologie und Infektionsimmunologie Nationales Konsiliarlabor für Dermatophyten Berlin Deutschland; ^8^ Kath. Kinderkrankenhaus Wilhelmstift Hamburg Hamburg Deutschland; ^9^ Charité – Universitätsmedizin Berlin, Korporatives Mitglied Freie Universität Berlin und Humboldt Universität zu Berlin Klinik für Dermatologie, Venerologie und Allergologie Berlin Deutschland; ^10^ Charité – Universitätsmedizin Berlin, Korporatives Mitglied Freie Universität Berlin und Humboldt Universität zu Berlin Klinik für Dermatologie, Venerologie und Allergologie, Division of Evidence Based Medicine (dEBM) Berlin Deutschland; ^11^ Klinik für Dermatologie, Universität Würzburg Würzburg Deutschland; ^12^ Universitätsklinikum Schleswig‐Holstein, Campus Kiel Klinik für Dermatologie, Venerologie und Allergologie Kiel Deutschland; ^13^ Main Medic All Health MVZ GmbH, Frankfurt am Main Frankfurt am Main Deutschland; ^14^ Sektion Kinderdermatologie, MUC iSPZ Hauner, LMU Zentrum für Entwicklung und komplex chronisch kranke Kinder München Deutschland; ^15^ HOCH Health Ostschweiz, Kantonsspital St. Gallen, Klinik für Dermatologie, Venerologie und Allergologie, St. Gallen Schweiz; ^16^ Büro für Dermatologie Neuhaus Deutschland; ^17^ Universitäts‐Hautklinik Tübingen Eberhard Karls Universität Tübingen Tübingen Deutschland; ^18^ labopart – Medizinische Laboratorien Dr. Gerber, Prof. Dr. Bühling, Prof. Dr. Nenoff, T. Löwe, E. von Rein Mölbis Deutschland

**Keywords:** antimykotische Therapie, mykologische Diagnostik, Tinea capitis, Antifungal therapy, Mycological diagnostics, Tinea capitis

## Abstract

Die unter Federführung der Deutschen Dermatologischen Gesellschaft e. V. (DDG) erstellte S1‐Leitlinie soll Ärzten eine strukturierte Entscheidungsgrundlage für die Versorgung von Patienten mit Tinea capitis bieten und zur Verbesserung der Diagnosesicherheit, Therapieadhärenz und Prävention beitragen.

Die Tinea capitis ist eine durch Dermatophyten verursachte Infektion der behaarten Kopfhaut, die überwiegend im Kindesalter auftritt und weltweit eine häufige dermatologische Erkrankung darstellt. In den letzten Jahren ist in Europa, begünstigt durch Migration, Tourismus und sozioökonomische Faktoren, ein verändertes Spektrum der auslösenden Dermatophyten zu beobachten, insbesondere durch eine Zunahme anthropophiler Erreger wie *Trichophyton tonsurans* und *Trichophyton violaceum*.

Therapeutisch ist eine Kombination aus systemischer und lokaler antimykotischer Behandlung angezeigt, eine alleinige topische Therapie ist in der Regel nicht ausreichend. Die Wahl des systemischen Antimykotikums richtet sich bei Kindern primär nach dem Erregerspektrum, wobei *Trichophyton*‐Infektionen bevorzugt mit Terbinafin (*off‐label*) und *Microsporum/Nannizzia*‐Infektionen mit Itraconazol (*off‐label*) oder Griseofulvin behandelt werden sollten. Bei erwachsenen Patienten ist das Mittel der ersten Wahl erregerunabhängig Terbinafin.

Darüber hinaus adressiert die Leitlinie Maßnahmen zur Unterbrechung von Infektionsketten, einschließlich der Mitbehandlung asymptomatischer Kontaktpersonen, hygienischer Maßnahmen sowie des Managements bei Ausbruchsgeschehen in Gemeinschaftseinrichtungen.

## EINLEITUNG

Die vorliegende Publikation stellt eine Kurzfassung der Leitlinie dar. Einige Kapitel sind in dieser Kurzversion nicht enthalten. Der Text der enthaltenen Kapitel ist zum Teil stark gekürzt. Die vollständige Langfassung der Leitlinie ist auf der Webseite der AWMF einsehbar: https://register.awmf.org/de/leitlinien/detail/013‐033

Diese S1‐Leitlinie stellt eine Aktualisierung der 2019 erschienenen deutschen Leitlinie dar.^1^ Es wurden die Ergebnisse einer Pubmed‐Recherche (Abruf August 2024) unter besonderer Berücksichtigung aktueller internationaler Leitlinien eingearbeitet, insbesondere der Leitlinien der European Society for Pediatric Dermatology 2010,^2^ sowie der British Association of Dermatologists 2014.^3^

### Definition

Die Tinea capitis (TC) ist eine durch Dermatophyten hervorgerufene und vor allem im Kindesalter auftretende Mykose der behaarten Kopfhaut.^2^, ^3^

### Erreger und Übertragung

*Microsporum (M.) canis* ist weltweit der häufigste zoophile Erreger, während *Trichophyton *(*T.) violaceum* und *T. tonsurans* die vorherrschenden anthropophilen Erreger der TC sind.^4^, ^5^, ^6^ Die Frequenz der letzteren hat weltweit in den letzten 20 Jahren zugenommen, wobei insbesondere sozioökonomische Faktoren als auch Tourismus und Migration eine Rolle spielen. Auch aktuelle epidemiologische Daten aus Europa zeigen eine solche Verschiebung, jedoch mit deutlichen regionalen Unterschieden.^4^, ^5^, ^6^, ^7^, ^8^, ^9^, ^10^, ^11^, ^12^, ^13^, ^14^, ^15^, ^16^, ^17^, ^18^, ^19^, ^20^, ^21^, ^22^, ^23^, ^24^, ^25^, ^26^, ^27^, ^28^, ^29^, ^30^ Auffallend ist ein stetiger Anstieg anthropophiler Erreger (*M. audouinii, T. tonsurans, T. violaceum, T. soudanense*) in West‐ und Nordeuropa,^7^, ^8^, ^9^, ^10^, ^11^, ^12^, ^13^ sowie in der Schweiz,^16^, ^17^ und auch in Deutschland,^31^ deren hohe Infektiosität sowie die Möglichkeit eines asymptomatischen Erregerstatus zu weiteren Infektionen und Kleinepidemien führen können.^4^, ^5^, ^6^

Neben den weiteren bei der TC bedeutsamen zoophilen Erregern *T. mentagrophytes, T. quinckeanum*,^32^, ^33^, ^34^, ^35^ und *T. verrucosum* hat auch *T. benhamiae* an Bedeutung gewonnen.^36^ Beim Kerion Celsi sind *M*. *canis* und *T*. *tonsurans* die häufigsten Erreger, aber auch andere Dermatophyten, wie *T. mentagrophytes* und *T. verrucosum* können ursächlich sein. ^37^, ^38^, ^39^ In Deutschland ist heute mit *T. tonsurans* nicht nur bei Migranten zu rechnen, sondern auch bei jugendlichen Kampfsportlern, insbesondere beim Ringkampf.^40^, ^41^ Aktuell wird ein vermehrtes Auftreten bei Jungen, männlichen Jugendlichen sowie bei jungen Männern ca. 2–3 Wochen nach Rasur von Bart oder Haarschnitt in sogenannten Barbershops beobachtet. Nachgewiesen wurden in einigen Fällen verunreinigte Friseurutensilien wie Rasiergeräte und Kämme, unzureichende Hygienemaßnahmen sowie Mikrotraumen.^42^, ^43^, ^44^, ^45^ *T*. *tonsurans* muss heute als „*Emerging Pathogen*“ vor allem bei der TC angesehen werden. Die Infektionszahlen sind seit 2020 deutlich angestiegen.^46^

### Befallsmuster und Klinik

Dermatophytosen der Terminalhaare (Trichomykosen) werden unter klinischen Gesichtspunkten und der mikroskopisch erfassbaren Befallsart in die Tinea im engeren Sinne, die Mikrosporie im engeren Sinne und den Favus (Tinea favosa) unterteilt.^47^, ^48^ Die TC ist die wahrscheinlich häufigste Dermatophytose im Kindesalter insbesondere zwischen dem 3. und 7. Lebensjahr, Säuglinge und Neugeborene sind selten betroffen.^49^, ^50^, ^51^, ^52^ Mit der Pubertät tritt eine TC selten auf. Als ein bedeutsamer Faktor wird hierbei eine fungistatische Aktivität der mit der Pubertät veränderten Sebumtriglyceride diskutiert.^53^

Trichomykosen entstehen durch Ausbreitung der Erreger vom Stratum corneum in Haarfollikel und ‐schaft bis zur Zone der Keratinisierung (Adamson‐Quaste). Mit dem Wachsen des Haars nach außen kommt es zur Verbreitung von Sporen. In Abhängigkeit von Erreger und Immunantwort resultiert die klinische Form, die von weitgehend aphlegmasischen Veränderungen mit nur leichter Schuppung bis zu tief infiltrierenden und abszedierenden Infektionen (Kerion) reichen kann. Schildchen‐förmige Krusten (Scutula), die zu größeren Massen konfluieren können, finden sich beim Favus (Tinea favosa). Das klinische Bild erlaubt aber keine sichere Zuordnung zum ursächlichen Erreger. Aus der (mikroskopisch erfassbaren) Form und Lage der Sporen zum Haar können sich aber Hinweise ergeben.

Am Haar wird eine Ektothrix‐Infektion, wobei der Pilz überwiegend in Form von Arthrosporen an der Oberfläche des Haarschaftes anhaftet, von einer Endothrix‐Infektion unterschieden, bei der der Erreger in den Haarschaft eindringt, ohne die Kutikula zu zerstören. Typische Erreger für die ektotriche Infektionsform sind *M. canis* und *M. audouinii* (kleine Sporen, „Mikrosporie“) sowie *T. mentagrophytes* und *T. verrucosum* (große Sporen). Die meisten anthropophilen Erreger (insbesondere *T. tonsurans, T. violaceum, T. soudanense*) verursachen eine endotriche Infektion des Haares. Das Haar ist bei intakter Kutikula mit Sporen angefüllt und aufgrund der Trichomalazie als kleiner schwarzer Punkt in der Follikelöffnung erkennbar („*black‐dot ringworm*“). Endothrix‐Infektionen durch anthropophile Erreger sind aufgrund der Lage der Sporen im Haarschaft oft nur wenig immunogen. Die Kopfhaut zeigt bei fehlender oder geringer Rötung häufig nur eine pityriasiforme Schuppung. Nicht selten resultiert ein asymptomatischer Überträgerstatus (in Risikopopulationen bis 15%; insbesondere bei Erwachsenen), der für die Ausbreitung der Infektion auf andere Personen bedeutsam sein kann.^54^ Dringen Dermatophyten an den Haarfollikeln in die Tiefe, kommt es zu follikulären Pusteln und zum Teil massiver eitriger Sekretion, was mit Allgemeinerscheinungen wie Fieber und Kopfschmerzen sowie Lymphknotenschwellung verbunden sein kann. Eine Fehldeutung als bakterieller Abszess ist nicht ungewöhnlich, sekundäre bakterielle Infektionen sollten aber nicht übersehen werden. Bei scheibenförmiger Ausprägung mit massiver eitriger Sekretion (wie der „Honig aus einer Wabe“) spricht man von Kerion (griechisch Honigwabe) Celsi (Aulus Cornelius Celsus, römischer Enzyklopädist und Medizinschriftsteller). Auslöser sind meist zoophile Dermatophyten wie *T. verrucosum, T. mentagrophytes, T. benhamiae, T. quinckeanum* oder die geophile *Nannizzia gypsea* (früher *M. gypseum*).^55^ In den letzten Jahren wurden aber gerade in urbanen Regionen auch entzündliche Ausprägungen von Endothrix‐Infektionen insbesondere durch *T. tonsurans* oder *T. violaceum* beobachtet.^56^

### Mikrosporie

Bei der Mikrosporie im engeren Sinne handelt es sich um eine nicht abszedierende, klinisch fast reaktionslose tiefe Follikulitis durch *M. audouinii* (anthropophil) oder *M. canis* (zoophil), ^57^ seltener durch andere *Microsporum/Nannizzia* Spezies (spp.) wie *M. ferrugineum* (anthropophil)^58^ oder *Nannizzia gypsea* (geophil). Betroffen sind fast nur präpubertäre Kinder. Eine entzündliche Reaktion fehlt meist völlig. Die glanzlosen Haare sind in den wie mit Mehl bestäubt erscheinenden oft kreisrunden, scharf begrenzten Herden kurz über dem Haarboden abgebrochen. Die Größe der Herde ist variabel, sie können einzeln oder multipel und dann zum Teil konfluierend vorkommen. Typisch bei der mikroskopischen Untersuchung von Haarstümpfen ist eine große Menge kleiner Sporen, die den Haarschaft umscheiden (Durchmesser 2–3 µm), weshalb die Erkrankung auch Mikrosporie genannt wird. Zeigt sich im Woodlicht eine gelbgrüne Fluoreszenz, so gilt die Diagnose einer *Microsporum/Nannizzia*‐Erkrankung als gesichert.

### Favus (Erbgrind)

Der Favus ist in Europa nur noch selten anzutreffen. Dennoch werden vereinzelt Fälle beschrieben, insbesondere aus Nordafrika und Tunesien. Auch in Deutschland tritt Favus durch Trichophyton schoenleinii weiterhin vereinzelt auf.^59^ Der Favus ist gekennzeichnet durch Myzelmassen enthaltende, schildchenförmige Schuppenkrusten, den Scutula, die sich im und um den Haarfollikel entwickeln, im Zentrum einen oder mehrere Haarschäfte umschließen und mit narbiger Alopezie (Pseudopeladezustand) abheilen. Ein „übler“ mäuseurinartiger Geruch komplettiert die Symptomatik. 95% der Erkrankten haben klinische Symptome, insbesondere Scutula, Ausbleichen der Haare, Atrophie und Vernarbung. Das Erkrankungsmaximum liegt bei Kindern im Alter von 6–10 Jahren (prädominant bei Jungen), die Erkrankung bildet sich aber mit der Pubertät nicht zurück.^60^ Sie kann lebenslänglich bestehen bleiben, die durchschnittliche Erkrankungsdauer beträgt fünf Jahre (10 Tage bis 59 Jahre). 7% der Tinea favosa Fälle zeigen auch einen Befall der übrigen Haut sowie der Nägel (Onychomycosis favosa).

Favus ist weniger ansteckend als andere Dermatophytosen, bedingt durch das Wachstum des Erregers innerhalb des Haarschafts (endothricher Favus). Andererseits befindet er sich hier auch in einer der Infektabwehr und Umwelteinflüssen nur schwer zugänglichen Nische. So kann der Erreger in epilierten Haaren bis zu 54 Monaten überdauern.

Die TC verursacht im Allgemeinen keinen dauerhaften Haarverlust. Eine irreversible Zerstörung des Haarfollikels mit vernarbender Alopezie (Pseudopeladezustand) ist durch eine destruierende Entzündungsreaktion insbesondere bei der TC profunda oder durch eine Infektion mit *T. schoenleinii* (Favus) möglich.

Bei der klinischen Verdachtsdiagnose TC soll neben einer gründlichen Anamnese eine Ganzkörperinspektion auf weitere Manifestationen einer Dermatophytose einschließlich einer Onychomykose erfolgen.^61^ Ferner können rezidivierende Manifestationen einer Dermatophytose beziehungsweise einer Onychomykose im Kindesalter Folge einer Autoinokulation sein. Umgekehrt kann von einer TC ausgehend eine Onychomykose der Fingernägel, seltener der Fußnägel entstehen. Diese Tinea unguium ist eine direkte Folge des Kratzens an der befallenen Kopfhaut. Die TC manifestiert sich beim Erwachsenen seltener, zeigt aber eine zunehmende Inzidenz.^3^, ^14^, ^19^ Das Krankheitsbild tritt häufiger bei Frauen als bei Männern auf, ursächlich werden u.a. hormonelle Störungen, Immunsuppression, Autoinokulation und Übertragung durch erkrankte Kinder diskutiert. Ungewöhnliche Klinik und Verläufe,^62^ die „*side‐cut*“ TC nach Rasur im Barbershop durch *T. tonsurans* ^42^, ^43^, ^63^, ^64^ und für die Erkrankung bei Kindern eher untypische Erreger wie *T. rubrum* ^65^ sind in der Diagnostik zu beachten.

Bei TC können Dermatophyten‐bedingte Mykide (Dermatophytide) als Ausdruck einer Spättyp‐Reaktion gegenüber dem Erreger eine große klinische Varianz aufweisen.^66^, ^67^ Kriterien für ein Dermatophytid sind: 1. Nachgewiesene, oft entzündliche Dermatophytose an einer tineafreien Lokalisation (hier TC); 2. Kein Nachweis von Pilz‐Elementen beziehungsweise ‐DNA in den dermatophytidbedingten Effloreszenzen; 3. Oft zunächst Verschlechterung unter Therapie mit systemischen Antimykotika (Freisetzung großer Mengen von Erregerantigen). Dies muss von einer Arzneimittelreaktion auf das systemisch verabreichte Antimykotikum abgegrenzt werden. 4. Abheilung nach Therapie der Dermatophytose.^68^, ^69^ Dermatophytide können grundsätzlich am Rumpf, an den Extremitäten und im Gesicht auftreten.^70^ Auch generalisierte Reaktionen sind möglich. Bei der TC beschrieben sind symmetrische disseminierte kleinpapulöse oder papulovesikulöse ekzemartige lichenoide Reaktionen mit follikulärer Akzentuierung meist am Stamm (Lichen trichophyticus), das Erythema nodosum sowie eine Erysipel‐ähnliche Dermatitis, psoriasiforme Veränderungen, das Erythema multiforme, sowie Erythema anulare centrifugum‐artige und urtikarielle Veränderungen.^66^, ^67^, ^71^, ^72^, ^73^, ^74^

### Differenzialdiagnose

Die Differentialdiagnose der TC ist sehr breit und umfasst alle Symptomatiken mit fleckförmigem Haarverlust, Schuppung oder Entzündung der Kopfhaut.^1^, ^3^, ^47^ Psoriasis capitis, das seborrhoische und das atopische Kopfekzem, ein Sekundärstadium der Syphilis (Alopecia areolaris specifica) sowie das Exsikkations‐Ekzem und die Pityriasis amiantacea („Tinea amiantacea“) sind gelegentlich schwierig von wenig entzündlichen Formen der TC zu unterscheiden. Die Alopecia areata zeigt im Allgemeinen keine Schuppung, aber gelegentlich Entzündung. Ausrufezeichenhaare müssen von den abgebrochenen Haaren bei der TC abgegrenzt werden. Lupus erythematodes, Lichen planopilaris und Trichotillomanie sind zu erwägen, besonders wenn auch Vernarbung eintritt. Entzündliche Formen der TC können als bakterielle Follikulitis, Abszesse (Furunkel, Karbunkel), Folliculitis decalvans oder als eosinophile pustulöse Follikulitis des Kleinkindalters fehlgedeutet werden. Zu den selteneren Differentialdiagnosen zählt das kutane T‐Zell Lymphom im Kindesalter.

## DIAGNOSTIK

Die Diagnose einer TC basiert auf klinischen und labormedizinischen Untersuchungen.^48^, ^75^, ^76^ Erstere bestehen im Wesentlichen aus der Anamnese (Verlauf, weitere Erkrankungsfälle in der Umgebung, Tierkontakte, Auslandsaufenthalte), der Inspektion (Befallsart, weitere klinische Manifestationen einer Dermatophytose wie Tinea corporis, Onychomykose), sowie gegebenenfalls der Untersuchung mittels UV‐Licht (∼365 nm; sogenanntes Wood‐Licht) und Auflichtmikroskopie. Labornachweise beruhen auf direkt‐mikroskopischen (im Nativpräparat), gegebenenfalls histologischen (insbesondere mit PAS‐Färbung) Untersuchungen sowie dem kulturellen oder PCR‐gestützten Erregernachweis.^77^ Der Erregernachweis ist bedeutsam für die Wahl des Antimykotikums sowie zur Aufdeckung von Infektionsketten. Besonders bei Epidemien und auch bei der Auffindung erkrankter Tiere kann die Wood‐Licht Untersuchung eine Hilfe sein. Zeigt sich eine gelb‐grüne Fluoreszenz, so ist die Diagnose einer *Microsporum*‐Erkrankung (zum Beispiel durch *M. canis*) gesichert. Allerdings ist die Sensitivität bei einer *Microsporum*‐Infektion des behaarten Kopfes nicht sehr groß und die Untersuchung damit für eine Ausschlussdiagnose nicht geeignet.

### Materialgewinnung

Voraussetzung für valide Untersuchungsergebnisse ist eine optimale Probenentnahme.^47^ Hierzu sollte steriles Instrumentarium verwendet werden. Bei Verwendung von Selektivnährmedien mit Cycloheximid und Antibiotika kann auf vorherige Desinfektion der Entnahmestelle verzichtet werden. Verdächtige Läsionen kann man klinisch oder gegebenenfalls mit Hilfe einer Wood‐Lampe oder trichoskopisch identifizieren. Bei entzündlichen tiefen Veränderungen sollen eventuell vorhandene Eiterkrusten mit der Pinzette entfernt und Haarstümpfe aus dem Rand des Krankheitsherdes gezupft werden. Diese lassen sich durch die stark entzündlichen Veränderungen (mit einer sterilen Pinzette oder einem Nadelhalter) leicht herausziehen. Bei wenig entzündlichen Läsionen können ausreichend Schuppen und Haare von der Kopfhaut entweder mit der stumpfen Seite eines Skalpells oder mittels der Bürsten‐ oder Abstrichmethode gewonnen werden. Bei der Bürstenmethode werden Haar und Haarboden mehrmals mit einer (sterilen) Kopfmassagebürste durchkämmt, die dann direkt leicht auf den Agar gedrückt wird, so dass an den Zinken/Borsten anhaftendes Material verimpft wird. Die Zahl der positiven Abdrücke erlaubt eine näherungsweise Quantifizierung.^78^

### Mikroskopie und Erregerbestimmung

Für die Direktmikroskopie („Nativpräparat”) wird das Material auf einem Objektträger mit 10 bis 20%iger Kalilauge (KOH) oder zu einer besseren Kontrastierung mit einem geeigneten Farbstoff (zum Beispiel 0,025% Kongorot in 5% Natriumdodecylsulfat (SDS)‐Lösung, Parker Tinte oder Chicago sky blue) überschichtet. Auf das Untersuchungsmaterial wird ein Deckgläschen gegeben und das Präparat in eine feuchte Kammer für 10–30 Minuten verbracht. Bei ca. 10‐ (Übersicht) und 40facher Objektiv‐ und 10facher Okularvergrößerung wird das Präparat unter dem Mikroskop durchgemustert. ^47^ Nachweis von (bei Dermatophyten farblosen) Hyphen und Sporen im Nativpräparat zeigt die Pilzinfektion an, kann aber keine verlässliche Auskunft über die Art des Erregers geben. Bei guter Präparation und eher kurzer Inkubation in KOH resp. plus Farbstoff, da sonst die Haarstruktur zu sehr geschädigt wird, lässt sich der ekto‐ vom endotrichen Pilzbefall der Haare unterscheiden. Auch die Eigenfarbe und die Form der Pilzelemente kann erkennbar sein, hilfreich zum Beispiel in der Abgrenzung zu manchen Schimmelpilzen. Die Sensitivität des Nativpräparates ist nicht sehr hoch, kann aber durch Zugabe optischer Aufheller (Calcofluor white®) und Beurteilung unter dem Fluoreszenzmikroskop gesteigert werden.^47^, ^79^

Zur Anzucht von Pilzen aus Haarwurzeln und Schuppenmaterial sind folgende Nährböden geeignet: ^79^ Sabouraud‐Glucose Agar mit 2 oder 4% Glucose, Kimmig‐Agar sowie ein Antibiotika‐ und Cycloheximid‐haltiger Agar (zum Beispiel Dermasel® Agar von Thermo Fisher Scientific, Schwerte, Germany) oder Dermatophyten Agar (zum Beispiel von Sifin, Berlin, Germany). Die Zusätze sind zur Unterdrückung eines Bakterien‐ und Schimmelpilzwachstums sinnvoll, welches sonst in einer raschen Überlagerung des langsamer wachsenden Dermatophyten resultieren kann. Zur Beimpfung sollten Schuppen und Haare auf den Agar verbracht und Haarstümpfe mit der Wurzel in den Nährboden „gepflanzt“ werden. Die Pilzkultur wird bei 26–28°C über drei bis vier, bei Verdacht auf eine *T. verrucosum‐, T. violaceum‐* oder *T. soudanense*‐Infektion bis zu sechs Wochen aufbewahrt und wöchentlich makroskopisch auf Pilzwachstum kontrolliert.^80^ Eine Inkubationstemperatur von 37°C beschleunigt das Wachstum von *T. verrucosum*, ist aber für andere Dermatophyten zu hoch. ^77^

Die Speziesidentifizierung erfolgt durch makroskopische Beurteilung der Thallusmorphologie und ‐farbe und eine mikroskopische Bestimmung von Makro‐ und Mikrokonidien sowie weiterer typischer Wachstumsformen wie zum Beispiel Chlamydosporen oder Spiralhyphen.

### Molekulare Methoden zum Erregernachweis bei TC

Molekulare Methoden wie konventionelle PCR mit anschließender Speziesidentifizierung über Sequenzierung oder den Einsatz spezifischer Sonden (ELISA‐Format, Microarray, Blot), der Realtime‐PCR oder der MALDI‐TOF MS (Matrix–Assistierte Laser–Desorption–Ionisierung Massenspektrometrie) sind hochsensitive und ‐spezifische Methoden, die zum einen zur Differenzierung eines Erregers aus der Primärkultur, andererseits aber auch (PCR‐Methoden) zum direkten Erregernachweis aus klinischem Material geeignet sind, was ein Ergebnis innerhalb von 24 Stunden ermöglicht. Mit MALDI‐TOF MS können die häufigen Dermatophyten aus der Pilzkultur heraus schnell und zuverlässig identifiziert werden, natürlich nur, wenn die dafür notwendige Datenbank vorliegt.^81^, ^82^, ^83^, ^84^, ^85^, ^86^ Einschränkend muss gesagt werden, dass insbesondere im *T*. *mentagrophytes*‐Komplex eine Unterscheidung der dort enthaltenen Spezies und Genotypen aktuell noch nicht möglich ist. ^87^ Dafür werden sowohl Inhouse‐Verfahren als auch zunehmend kommerziell verfügbare Testsysteme (Kits) eingesetzt.^88^, ^89^, ^90^ Derzeit werden mindestens 5 kommerzielle Testsysteme angeboten, darunter ein Multiplex‐PCR‐Microarray zum gleichzeitigen Nachweis von mindestens 23 Dermatophyten und sechs zusätzlichen relevanten Nicht‐Dermatophyten‐Pilzen (je 3 Hefen bzw. Schimmelpilze) mittels Hybridisierung nach Amplifikation der Pilz‐DNA in nur einem Reaktionsschritt. Damit sind molekulare Techniken deutlich schneller (24–48 Stunden verglichen mit 2–6 Wochen bei kulturellen Methoden) und sensitiver als Nativpräparat und Kultur.^73^ Seltene oder neu beschriebene Spezies können durch diese Tests meist jedoch nicht erkannt oder differenziert werden. Falsch negative Ergebnisse sind auch hierbei möglich, was unter anderem an der nicht homogenen Verteilung der Pilzelemente im Untersuchungsmaterial liegt (Vorkommen der Pilzelemente zumeist in Nestern). Daher sollte das Ausgangsmaterial – Kopfschuppen und Haarwurzeln, gegebenenfalls Abstriche – immer in ausreichender Menge gewonnen und entsprechend angesetzt werden. Gleichfalls muss man den PCR‐Befund immer auch kritisch dem klinischen Bild gegenüberstellen, weil zum Beispiel aufgrund der hohen Empfindlichkeit der Methodik immer auch DNA‐Verunreinigungen am Entnahmebesteck, im Entnahmeraum oder Laborbereich die Probe kontaminieren können (falsch positive Ergebnisse). Bei sorgfältiger Arbeit besonders im molekularbiologischen Labor ist diese Kontaminationsmöglichkeit jedoch nahezu auszuschließen.

Inwieweit sich die PCR neben dem schnelleren Erregernachweis auch zur Bewertung des Therapieverlaufs bei der TC eignet, ist zur Zeit nicht geklärt und erfordert weitere Untersuchungen. Eigene Erfahrungen zeigen, dass nach kombinierter topischer und systemischer antimykotischen Therapie einer TC durch *T*. *tonsurans* auch nach klinischer Ausbehandlung die Kultur noch bis zu zwei Monaten Wachstum zeigen, die PCR sogar noch nach drei Monaten positiv ausfallen kann. Dem entspricht auch das Fazit der kürzlich publizierten Studie aus der Schweiz, bei der die PCR aufgrund ihrer zu hohen Empfindlichkeit als nicht geeignet zur Therapiekontrolle bei TC angesehen wurde.^91^ Es gab eine deutlich plausiblere Korrelation des Therapieerfolges zur Pilzkultur. Weitere Untersuchungen zum Wert der PCR zur Verlaufskontrolle und zum Therapieerfolg sind dringend erforderlich.

### Histologie

Bei vorab behandelten Patienten mit wiederholt unplausiblen negativen mykologischen Untersuchungsergebnissen kann eine histologische Untersuchung von Biopsien in Kombination mit einer Pilzfärbung (PAS, Grocott‐Gomori‐Versilberungstechnik, Calcofluor white) und gegebenenfalls einer PCR hilfreich sein.^92^, ^93^ Eine histologische Untersuchung ist vor allem bei der TC profunda oder auch beim Favus,^59^ vorteilhaft, da hier in Schuppenmaterial Pilzelemente sehr spärlich vorhanden sein können. Außerdem lassen sich mittels PCR‐Diagnostik auch in formalinfixiertem Biopsiematerial Dermatophyten auf Speziesebene identifizieren.^93^

### Dermatoskopie/Trichoskopie

Nach aktuellen Studienergebnissen erlaubt die Trichoskopie als ein Hilfsmittel in der Diagnostik der TC eine einfache, rasche, nicht‐invasive Diagnosestellung und hat ihren Wert vor allem auch unter Bedingungen, bei denen eine mykologische Untersuchung nicht verfügbar ist. Sie ermöglicht anhand charakteristischer Befunde auch Hinweise auf den Infektionstyp (ektothrix, endothrix) und damit auch auf die Wahl des einzusetzenden Therapeutikums, aber keinen definitiven Erregernachweis.^75^, ^94^, ^95^, ^96^

### Resistenztestung

Da eine verstärkte Resistenzentwicklung bei Dermatophyten bisher nicht nachgewiesen werden konnte, ist eine routinemäßige Resistenztestung von Dermatophyten, die bei TC isoliert werden, derzeit nicht angebracht.^97^, ^98^

## THERAPIE

Die Therapie der TC verfolgt im Wesentlichen vier Ziele, die in einem Behandlungsplan mit allen Beteiligten abzustimmen sind:^1^, ^2^, ^3^, ^99^

1. Klinische und mykologische Heilung mit schnellstmöglicher sicherer Erregerelimination (Nativpräparat und Kultur negativ);
2. rasche Beseitigung der Symptome;
3. Vermeidung eines bleibenden Haarverlustes;
4. Verhinderung weiterer Übertragung beziehungsweise Unterbrechung von Infektionsketten.

Eine TC sollte immer systemisch und zusätzlich lokal behandelt werden, was meist ambulant gelingt. Eine Hospitalisierung wegen einer TC ist nur selten (schwere Fälle) und fast nur beim Kerion Celsi erforderlich. Chirurgische Interventionen bei Abszedierungen am Capillitium mit der Differentialdiagnose „Mykose“ ohne abgeschlossene mykologische Diagnostik sind kontraindiziert.^100^, ^101^

### Behandlungsbeginn

Eine gezielte Therapie setzt eine exakte Diagnostik mit Pilznachweis im Nativpräparat und kulturell oder mittels PCR‐gestützter Methoden voraus. Ausnahmen gelten für Anbehandlung bei begründetem Verdacht in schweren Fällen (auch ohne Abwarten einer „Schnelldiagnostik“ mittels PCR und auch bei uneindeutigen mikroskopischen Befunden) sowie bei Verdacht auf Infektion mit resistenten Stämmen bei hohem Übertragungsrisiko in Einzelfallabwägung.^3^ Zur Unterbrechung einer Infektionskette sollte in erster Linie die schnellstmögliche Diagnostik herangezogen werden (Direktpräparat + PCR). Eine Kultur wäre optimal parallel zur PCR.

Die Identifizierung des Erregers ist mit entscheidend für Wahl, Art und Dauer der Gabe und Dosis des Antimykotikums. Bei einer systemischen Therapie sollten das Alter des Patienten, bei Kindern zusätzlich das Körpergewicht sowie mögliche Wechselwirkungen mit weiteren Medikamenten in die Therapieplanung eingehen. Bei Nachweis einer zoophilen Art sind für die Übertragung in Frage kommende Tiere veterinärmedizinisch zu untersuchen, auf Pilze auch ohne Vorliegen von Infektionszeichen zu testen und im positiven Fall antimykotisch zu behandeln. Weiterer Kontakt der Tiere zum Patienten und seinem Umfeld ist zu verhindern.

### Topische Therapie

Eine alleinige topische Therapie ist für die Behandlung der TC in der Regel nicht ausreichend.^1^, ^2^, ^3^ Topische Präparate sollten jedoch immer zusätzlich eingesetzt werden. Sie unterstützen durch die Erregerabtötung auch außerhalb der von systemischen Antimykotika erreichbaren Lokalisationen mit Reduktion der Sporen, die Infektiosität, verkürzen die Dauer einer systemischen Therapie und sind Mittel der ersten Wahl zur Behandlung von asymptomatischen Überträgern mit geringer Sporenlast.^78^, ^102^, ^103^, ^104^ Zur topischen Behandlung der befallenen Kopfhaut eignen sich bestimmte Azole, Amorolfin und Ciclopiroxolamin, letzteres vorzugsweise als Lösung. Eine Alternative ist Terbinafin‐haltige Creme. Die Cremes und Lösungen werden zweimal täglich appliziert, alternativ können zwei unterschiedliche Wirkstoffe morgens und abends im Wechsel zum Beispiel Ciclopiroxolamin und Clotrimazol, eingesetzt werden.^105^ Zusätzlich sollten antimykotisch wirksame Shampoos und/oder Lösungen mit Selen(di)sulfid, Ketoconazol, Clotrimazol, Ciclopiroxolamin oder Pirocton‐Olamin verwendet werden. Shampoos sollten mindestens über fünf Minuten zweimal wöchentlich über 2–4 Wochen appliziert werden. Eine Beschränkung der Anwendungsdauer eines Antimykotikums ist nicht sinnvoll. Dieses sollte nur nach klinischem und mykologischem Ansprechen in seiner Anwendungsdauer limitiert werden. Insbesondere im Säuglings‐ und Kleinkindalter sind besondere Hinweise zur Anwendung der topischen Antimykotika gemäß Fachinformation zu beachten.

Während die Präparate in der Behandlung der TC bei Kindern bis zum 12. Lebensjahr in Deutschland erstattungsfähig sind, müssen die Kosten für die Mitbehandlung asymptomatisch Infizierten sowie von Kontaktpersonen von diesen selbst getragen werden. Der Kostenfaktor dürfte daher für die Compliance der Patienten eine Rolle spielen. Dies ist von Bedeutung, da die Mitbehandlung asymptomatisch Erkrankter besonders bei Infektionen durch anthropophile Erreger wie *T. tonsurans* oder *M. audouinii* zur Eindämmung der Infektion essentiell ist.^1^, ^2^, ^3^ Oft handelt es sich dabei um Ausbruchssituationen, in denen unter Umständen auch das Gesundheitsamt für die Kostenübernahme in Betracht kommen kann.

### Systemische Therapie

Prinzipiell wirken systemische Antimykotika bei Vorliegen einer endotrichen (zum Beispiel durch *Trichophyton* *spp*.) deutlich besser als bei einer ektotrichen Infektion (zum Beispiel *M. canis*). Grundsätzlich sollte sich die antimykotische Therapie immer nach dem nachgewiesenen Erreger richten (Tabelle 1).^104^, ^106^, ^107^, ^108^, ^109^, ^110^, ^111^, ^112^, ^113^, ^114^, ^115^, ^116^, ^117^, ^118^

**TABELLE 1:** Auswahl des oralen Antimykotikums in Abhängigkeit vom isolierten Erreger zur Behandlung der TC im Kindesalter (vor der Pubertät).

*Trichophyton mentagrophytes, benhamiae, tonsurans, violaceum, soudanense* und andere *Trichophyton* spp.	Terbinafin
*Microsporum canis, M. audouinii, M. ferrugineum*	Itraconazol oder Griseofulvin (nicht mehr verfügbar)*
*Nannizzia gypsea*	Itraconazol oder Griseofulvin (nicht mehr verfügbar)*

Griseofulvin ist in Deutschland, Österreich und der Schweiz nicht mehr im Handel, wird aber international insbesondere bei *Microsporum/Nannizzia*‐Infektionen weiterhin empfohlen und ist das einzige in Deutschland bei Kindern mit TC zugelassene Antimykotikum.

Eine TC durch *Trichophyton*‐Arten sollte vorrangig mit Terbinafin, eine TC durch *Microsporum/Nannizzia spp*. mit Itraconazol behandelt werden (Tabelle 1).^1^, ^2^, ^3^, ^105^ Griseofulvin ist bei den letztgenannten Dermatophyten‐Gattungen bei TC im Kindesalter gegenüber Itraconazol gleichwertig. Da alle Griseofulvin‐Präparate jedoch seit 2018 in Deutschland und vielen anderen europäischen Ländern (Belgien, Griechenland, Portugal, Türkei, Österreich, Schweiz) nicht mehr im Handel sind und umständlich über die Internationale Apotheke (Einzelimporte nach § 73 AMG) bezogen werden müssen, sind in diesen Ländern die Verordnungen mit diesem Mittel stark zurückgegangen.^105^

Ein Therapiebeginn vor einer endgültigen Erregeridentifikation sollte immer die lokale Epidemiologie und den wahrscheinlichsten Erreger berücksichtigen.

Aus arzneimittelrechtlichen und pharmakokinetischen Gründen muss bei der Behandlung zwischen Erwachsenen und Kindern unterschieden werden.^1^ Für die orale Behandlung Erwachsener kommen die Antimykotika Terbinafin, Itraconazol oder Fluconazol in Frage, die zur Behandlung von “Dermatomykosen (zum Beispiel Tinea)” zugelassen sind (Tabelle 2). Für Kinder ist derzeit nur Griseofulvin zugelassen.

**TABELLE 2:** Orale Behandlung der TC Erwachsener

Präparat	Dosierung pro Tag	Behandlungsdauer^1^
Itraconazol	100–200 mg^2^ einmal täglich direkt nach einer Hauptmahlzeit	4 Wochen
SUBA‐(Super‐Bioavailability) Itraconazol	50 mg 2 x täglich (*off‐label*)	4 Wochen
Fluconazol	Fluconazol 50 mg einmal täglich, alternativ 150 mg einmal pro Woche	4–7 Wochen 4–8 Wochen
Terbinafin	250 mg einmal täglich	4–6 Wochen

^1^ Die angegebenen Behandlungszeiten sind Anhaltspunkte,^105^ die individuellen Zeiten richten sich nach dem klinischen Bild und dem Ausfall der mykologischen Untersuchung, die ab der 4. Behandlungswoche im 4‐wöchigen Abstand durchgeführt werden sollte. Ziel ist die mykologische Heilung. Eine zusätzliche Lokalbehandlung (siehe unten) ist auch bei Erwachsenen unverzichtbar. Eine Heilung ist bei Erwachsenen schneller als bei Kindern zu erwarten

^2^ Laut Herstellerangaben wird eine Dosis von 100–200 mg täglich empfohlen.

Fluconazol ist, wie auch Terbinafin und andere Azole, in Deutschland für die Behandlung von Dermatophyten‐Infektionen von Kindern generell nicht zugelassen (*off‐Label*). Daher ist auch Fluconazol, so wie die beiden anderen oralen Antimykotika Terbinafin und Itraconazol, bei Kindern nur als individueller Heilversuch laut AMG anwendbar (Tabelle 3).

**TABELLE 3:** Internationale Empfehlungen zur Behandlung der TC bei Kindern.^1^, ^2^, ^3^, ^104^, ^106^, ^107^

Präparat	Dosierung	Behandlungsdauer bei *Trichophyton spp* ^5^	Behandlungsdauer bei *Microsporum/ Nannizzia spp* ^5^
Itraconazol^1^, ^2^	5 mg/kg KG, 1 x täglich mit Hauptmahlzeit; oder bei < 20 kg KG 50 mg/Tag bei > 20 kg KG, 100 mg/Tag Unter 10 kg KG streng gewichts‐adaptiert geben.	4 Wochen	6 Wochen
Fluconazol^1^, ^3^	3–6 mg/kg KG 1x täglich	3–4 Wochen 4–8 Wochen	6–8 Wochen
Terbinafin^1^	< 20 kg KG 62,5 mg/Tag 20–40 kg KG 125 mg/Tag > 40 kg KG 250 mg/Tag	4 Wochen	8–12 Wochen Bedeutung bei der Behandlung dieser Erreger umstritten
Griseofulvin ultramikronisiert^4^	10–15 mg/kg KG 1x täglich (bei *M*. *canis* höhere Dosierung von 20–25 mg/kg KG) in ein bis zwei Einzeldosen täglich mit der Hauptmahlzeit	6–8 Wochen	8–12 Wochen

^1^ Präparate für Kinder nicht zugelassen, in Deutschland nur im Rahmen eines individuellen Heilversuches einsetzbar. Daten für eine Gabe von Terbinafin bei Kindern unter 10 kg Körpergewicht (unter 1–2 Jahren) sind nur in Einzelfällen publiziert.^121^

^2^ Tageshöchstdosis unabhängig vom Körpergewicht 100 mg/d. Eine Saftformulierung ist seit 1.2.2024 nicht mehr verfügbar. Alternativ kann nach Rücksprache mit dem jeweiligen Hersteller eine dem Körpergewicht entsprechende Menge einer 100 mg Kapsel in der Apotheke als Suspension umgefüllt werden. Bei kleineren Kindern ist eine Saft‐Formulierung eines anderen Wirkstoffes zu bevorzugen.

^3^ Fluconazol ist in Deutschland für die Behandlung von Dermatophyten‐Infektionen von Kindern generell nicht zugelassen. Nach Einwilligung der Erziehungsberechtigten ist ein individueller Heilversuch jedoch gerechtfertigt.

^4^ Generell zugelassen für Kinder. Säuglinge und Kleinkinder bis zu 2 Jahren nur in begründeten Ausnahmefällen. Nicht für Neugeborene verwenden. Griseofulvin in Deutschland außer Handel.

*durchschnittliche Mindestdauer, Ziel = negativer Pilznachweis (Kultur/PCR)

^5^ Die angegebenen Behandlungszeiten sind Anhaltspunkte, die individuellen Zeiten richten sich nach dem klinischen Bild und dem Ausfall der mykologischen Untersuchung, die ab der 4. Behandlungswoche im 2–4‐wöchigen Abstand durchgeführt werden sollte. Oft sind wesentlich längere Behandlungszeiträume von 3 bis 4 Monaten notwendig, nicht nur beim Kerion Celsi, sondern auch zum Beispiel bei besonders trockenen hyperkeratotischen Formen der TC.^105^

Wenn Griseofulvin nicht zur Anwendung kommen soll, sind neben einer ausführlichen Aufklärung des/der Erziehungsberechtigten über mögliche Risiken und Nebenwirkungen, potenzielle unbekannte Nebenwirkungen und schwerstmögliche Verläufe, die fehlende Zulassung des verordneten Medikamentes für die Altersgruppe, aber auch Vorteile der *Off‐Label*‐Therapie gegenüber dem einzigen in Deutschland zugelassenen Medikament Griseofulvin und gegebenenfalls anderen Antimykotika beziehungsweise der Nicht‐Behandlung neben einer adäquaten Dokumentation darüber zwingend erforderlich.^119^ In Österreich und der Schweiz ist Terbinafin zur Behandlung im Kindesalter ab dem 2. Lebensjahr zugelassen. Somit erfolgt der Einsatz der neueren Antimykotika bei Kindern zurzeit immer im Rahmen eines individuellen Heilversuches gemäß AMG. Eine Vielzahl von Studien zeigt aber, dass die neueren Antimykotika Terbinafin und Itraconazol höhere Ansprechraten, eine größere Therapiesicherheit und eine höhere Kosteneffektivität als Griseofulvin haben.^104^, ^106^, ^107^, ^108^, ^109^, ^110^, ^111^, ^112^, ^113^, ^114^, ^115^, ^116^, ^117^, ^118^, ^120^

### Ursachen des unbefriedigenden Ansprechens bei Kindern

In der Studie von Ghannoum et al.^97^ zeigte Terbinafin in vitro eine starke antimykotische Wirksamkeit gegenüber 301 Dermatophytenisolaten von Patienten mit TC weltweit, darunter *Microsporum canis* (n = 94) und *M. audouinii* (n = 19). Die ermittelten Ergebnisse spiegeln die klinische Realität insofern ungenügend wider, als systemisch verabreichtes Terbinafin bei Kindern mit *Microsporum*‐Infektionen aufgrund geringer Heilungsraten nicht Mittel der 1. Wahl ist. Die Gründe dafür liegen nach den Daten von Ghannoum et al. nicht in einer verminderten in vitro Empfindlichkeit oder Resistenz der Erreger. Das unbefriedigende Ansprechen bei Kindern hat seine Ursache eher in anatomischen und physiologischen Besonderheiten bei Kindern vor der Pubertät (trockene Haut, fehlende Fett‐ und Fettsäurebildung der Talgdrüsen) sowie im Wirkungsmechanismus sowohl der Azole als auch der Allylamine auf die Pilzzelle.^122^

### Behandlungsstrategie bei Vorliegen einer TC und Follow‐Up^1^, ^2^, ^3^, ^123^

1. Bei positivem Ergebnis des Direktpräparates, und/oder der Kultur/PCR und/oder der Woodlicht‐Testung Beginn der kombinierten systemischen und lokalen antimykotischen Therapie zunächst einheitlich über vier Wochen. Auswahl des Präparates je nach Anamnese/ Epidemiologie, Ergebnis der mykologischen Diagnostik (Präparat, Kultur und PCR).
2. Vier Wochen nach Beginn der Therapie erneute komplette mykologische Diagnostik (mit PCR soweit verfügbar). Bis zum Vorliegen des Kulturbefundes (maximale Inkubationsdauer vier Wochen) bei deutlicher Besserung des klinischen Befundes und immer bei sichtbar nachwachsenden Haaren Unterbrechung der systemischen Behandlung unter Fortführung der lokalen Therapie (gegebenenfalls Absicherung mittels Trichoskopie). Ansonsten sollten beide Therapieformen fortgeführt werden.
3. Falls die Kultur (und gegebenenfalls die PCR) weiterhin positiv ist, die topische und systemische Therapie für 2–4 weitere Wochen fortführen (bis zur nächsten mykologischen Untersuchung).
4. Wiederholung dieses Vorgehens (Punkte 2–3), bis der Kulturbefund negativ ist. Die mykologische Heilung ist Ziel und Endpunkt der Behandlung und richtet sich folglich nach den individuellen Verläufen der Patienten.

Kontrollen der Laborwerte: Traditionell wurden unter systemischer antimykotischer Therapie Laborkontrollen der Leberwerte (ASAT, ALAT, GGT) durchgeführt. Der Nutzen dieser Maßnahmen ist fraglich, da insbesondere schwere hepatotoxische Veränderungen innerhalb eines sehr kurzen Zeitfensters auftreten und nur schwer mit einer Routinekontrolle nach 4–6 Wochen zu erfassen sind.^124^, ^125^, ^126^. Andererseits ist von allen Substanzgruppen (wenn auch mit unterschiedlicher Frequenz/ Stärke) bekannt, dass Störungen der Leberfunktion auftreten können, in seltenen Fällen allerdings auch schwerwiegende bis zu letalem Ausgang. Aus diesen Gründen sind fokussierte Aufklärung und genaue Kenntnis des individuellen Status des Patienten sowie ein gewissenhaftes gesamt‐klinisches Monitoring nicht nur des Haarstatus von besonderer Bedeutung im Verlauf der nicht selten monatelangen antimykotischen Behandlung.

Folgendes Vorgehen wird daher empfohlen (Tabelle 4).

**TABELLE 4:** Laborkontrollen unter systemischer antimykotischer Therapie (Allylamine, Azole, Griseofulvin).

**Patienten mit vorbekannten Einschränkungen der Leberfunktion, hepatotoxischer Co‐Medikation oder sonstigen die Leberfunktion beeinträchtigender Komorbidität**	ASAT, ALAT und gGT vor Therapiebeginn, nach 2–4 Wochen, danach individuell je nach Verlauf
**Bei Patienten ohne Risikofaktoren**	Aktuell kein Konsens bzgl. Notwendigkeit von Laborkontrollen. Es sollte individuell je nach Dosierung, Therapiedauer und gegebenenfalls weiteren Faktoren gemeinsam mit dem Patienten entschieden und dokumentiert werden.
Patienten bzw. deren Erziehungsberechtigte sind im Rahmen der Aufklärung auf die möglichen Nebenwirkungen und insbesondere auf Symptome einer möglichen Leberschädigung dokumentiert hinzuweisen. Dies ist speziell bei den nicht zugelassenen Pharmaka bei der *Off‐Label*‐Therapie auch für den Therapeuten von hoher rechtlicher Relevanz. Beim Auftreten wegweisender gastrointestinaler Symptome sollte die Therapie zunächst unterbrochen, der Patient eingehend auch labormedizinisch abgeklärt werden. Auch bei schwereren immunologischen Unverträglichkeitsreaktionen kann eine Unterbrechung oder ein Absetzen der Medikation erforderlich werden. Voraussetzung einer sicheren Behandlung ist die regelmäßige Behandlungskontrolle (Visiten) und die Möglichkeit der Kontaktaufnahme bei Verdacht auf unerwünschte Wirkungen durch den aufgeklärten Patienten.	

### Zusätzliche Maßnahmen

#### Ausschluss von Schule und Kindergarten

Nach Sichtung der Datenlage und eigenen Erfahrungen der Leitlinienautoren, können Kinder, die eine geeignete systemische und adjuvante topische Therapie erhalten, die Schule oder den Kindergarten wieder besuchen. Bei hochkontagiösen anthropophilen Erregern (*T. violaceum, T. soudanense*, gegebenenfalls *T. tonsurans, M. audouinii*) kann eine einwöchige Besuchskarenz erwogen werden. Die anthropophilen Pilze sind infektiöser und kontagiöser, und sie verbreiten sich von einer eher trocken‐schuppenden TC leicht zu anderen Kindern. Unabhängig von dieser Regel sollten selbstverständlich Kinder mit eitrig‐abszedierender TC und Kerion Celsi so lange zu Hause bleiben, bis die Wundheilung abgeschlossen ist.

Ein asymptomatischer Überträgerstatus kann zur Persistenz von Infektionsketten beitragen. Bei anthropophilen Erregern wird eine Untersuchung und auch bei fehlender Klinik die topische Behandlung aller Familienmitglieder und engen Kontaktpersonen, bevorzugt mit antimykotisch wirksamen Shampoos, empfohlen. Bei zoophilen Erregern ist in veterinärmedizinischer Kooperation ein Screening und gegebenenfalls die Behandlung der Tiere empfehlenswert.

#### Haarrasur/Tragen einer Kopfbedeckung

Die Zeit bis zum Erlöschen der Infektiosität unter der Behandlung, und das gilt für alle Formen der kindlichen TC, ist auch von der Länge der infizierten Haare abhängig. Ein Zurückschneiden bzw. die Rasur der Haare kann die Behandlungsdauer mit einem systemischen Antimykotikum erheblich verkürzen. Durch die topische Kombinationstherapie mit hochwirksamen, fungizid wirkenden Antimykotika ist dieses Vorgehen ebenso wie die Empfehlung zum Tragen einer Kopfkappe jedoch nicht mehr erforderlich.

### Screening innerhalb der Familie

Insbesondere Erkrankungen durch anthropophile Erreger wie *T. tonsurans, T. violaceum, T. soudanense* und *M. audouinii* sind sehr ansteckend. Alle Familienmitglieder und Kontaktpersonen sollten hinsichtlich Symptomen befragt und untersucht werden.^127^ Mehr als 50% der Familienmitglieder einschließlich der Erwachsenen können betroffen sein, oft auch in einem asymptomatischen Überträgerstatus.^78^ Eine Nicht‐Behandlung symptomatischer oder asymptomatischer Familienmitglieder kann zu hohen Rezidiv‐Raten führen. Eine mykologische Untersuchung und gegebenenfalls Behandlung aller, auch asymptomatischer Familienmitglieder und enger Kontaktpersonen wird daher empfohlen.

Zusammenfassend wird nach Sichtung der Datenlage und eigenen Erfahrungen der Leitlinienautoren die Gabe systemischer Antimykotika bei Kontaktpersonen nur dann empfohlen, wenn tatsächlich Zeichen einer manifesten TC bestehen. Bei asymptomatischen Kontaktpersonen sollte eine topische antimykotische Therapie bevorzugt mit antimykotisch wirksamen Shampoos durchgeführt werden. Shampoos sollten mindestens über fünf Minuten zweimal wöchentlich über vier Wochen appliziert werden. Um die Compliance sicherzustellen, sollte über die Rationale dahinter eingehend aufgeklärt werden.^127^ Wiederholte mykologische Kontrollen (alle vier Wochen) sind bei symptomatischen Patienten erforderlich, um den Erfolg der Therapie sicher zu stellen.

### Vorgehen bei epidemischem Auftreten/Meldepflicht

Dermatophyteninfektionen wie die TC sind gemäß IfSG § 34 grundsätzlich nicht meldepflichtig. Im Rahmen der Regelungen für Gemeinschaftseinrichtungen besteht eine Meldepflicht jedoch dann, wenn zwei oder mehr gleichartige Erkrankungen auftreten, bei denen ein epidemischer, zeitlicher und räumlicher Zusammenhang wahrscheinlich ist oder vermutet wird und wenn dies auf eine schwerwiegende Gefahr für die Allgemeinheit hinweist.

### Behandlung kontaminierter Gegenstände und des Inventars

Vitale Sporen anthropophiler Spezies wurden von Haarbürsten und Kämmen isoliert.^128^, ^129^ Deshalb sind Utensilien der persönlichen Hygiene wie Kämme, Haarbürste, Rasierapparat, Handtücher, Waschlappen, Bettwäsche, Schals, Kopfbedeckungen, Plüschtiere und Spielgeräte bei Verdacht auf Pilzbefall immer zu desinfizieren beziehungsweise zu entsorgen oder durch Einmalutensilien zu ersetzen.^1^, ^130^ Einer geeigneten Desinfektion müssen aber auch Einrichtungsgegenstände wie Gardinen und Möbel/Polstermöbel unterzogen werden, wenn eine Kontamination durch Pilzelemente wahrscheinlich ist wie in Kindergärten oder bei Haustieren, die Auslöser für TC Infektionen mit zoophilen Erregern sein können. Bei zoophilen Erregern sollten Haustiere veterinärmedizinisch untersucht und behandelt werden. Haustiere müssen nicht erkrankt sein, sondern können asymptomatische Überträger für die entsprechenden Erreger darstellen. ^36^, ^131^

### Einsatz von Glukokortikoiden

Nach Ansicht einiger Autoren beruht die Ausbildung einer maximal inflammatorischen TC (Kerion) auch auf der Ausbildung einer starken Immunreaktion vom Spättyp auf den Erreger, kenntlich auch durch die häufige Ausbildung von Mykiden.^68^, ^69^ Daher wurde in der Literatur auch der Einsatz oraler oder intraläsionaler Glukokortikosteroide empfohlen, um die Heilung zu beschleunigen und das Risiko einer bleibenden Alopezie zu minimieren.^132^, ^133^ Einer Verstärkung der Immunreaktion beziehungsweise des Juckreizes durch eine hochpotente antimykotische Monotherapie und raschen Erregerzerfall bei inflammatorischen TC‐Formen kann mit einer topischen Kombinationstherapie aus Glukokortikosteroiden und Antimykotika initial für einen begrenzten Zeitraum von etwa sieben Tagen begegnet werden.^132^, ^133^

### Therapieversagen

Gründe für ein Therapieversagen können sein:

1. Fehlende Compliance – besonders bei langen Behandlungszeiten,^1^, ^2^, ^3^
2. Unterdosierung (insbesondere zu geringe Dosis über zu kurze Behandlungsdauer oder auch verminderte Wirksamkeit der oralen Antimykotika durch Co‐Medikation) ^134^, ^135^
3. Relative Resistenz des Erregers (keine ausreichenden Wirkstoffkonzentrationen am Wirkort) ^122^
4. Resistenz des Erregers (insbesondere. *T*. *mentagrophytes* ITS Genotyp VIII / *T. indotineae* durch Mutationen des Squalenepoxidase‐Gens).^105^, ^136^, ^137^, ^138^, ^139^, ^140^
5. Reinfektion
6. Grunderkrankungen des Betroffenen, die zu einer eingeschränkten Immunantwort führen.

Bei ausbleibender klinischer Besserung ist es wichtig sicherzustellen, dass die gewählte antimykotische Therapie für den nachgewiesenen Erreger adäquat ist (Tabelle 1). Im anderen Fall sind die Optionen:

1. Die Dosis bzw. die Therapiedauer des ursprünglichen Antimykotikums steigern
2. Das Antimykotikum zu wechseln (Tabelle 1, insbesondere auch bei Nachweis eines unerwarteten Erregers).

## DANKSAGUNG

Open access Veröffentlichung ermöglicht und organisiert durch Projekt DEAL.

## INTERESSENKONFLIKT

Siehe Langfassung der Leitlinie: https://register.awmf.org/de/leitlinien/detail/013‐033
